# Microbial Primer: LuxR-LuxI Quorum Sensing

**DOI:** 10.1099/mic.0.001343

**Published:** 2023-09-01

**Authors:** Martin P. Soto-Aceves, Stephen P. Diggle, E. Peter Greenberg

**Affiliations:** ^1^​ Department of Microbiology, University of Washington School of Medicine, Seattle, WA, USA; ^2^​ Center for Microbial Dynamics and Infection, School of Biological Sciences, Georgia Institute of Technology, Atlanta, GA, USA

**Keywords:** quorum sensing

## Abstract

Quorum sensing is a term describing bacterial cell-to-cell communication systems for monitoring and responding to changes in population density. This primer serves as an introduction to the canonical LuxR-LuxI-type quorum sensing circuits common to many species of Gram-negative bacteria. Quorum sensing can synchronize behaviours across a community. Different species employ quorum sensing strategies to control specific behaviours such as bioluminescence, virulence factor production, secondary metabolite production, and biofilm formation.

## The discovery of LuxR-LuxI quorum sensing

We have known for almost 30 years that there is a genetic regulatory circuit conserved among many species of *Proteobacteria *, the so-called LuxR-LuxI type quorum sensing (QS) circuit. This type of circuit was first described to control light production in the marine bacterium *Vibrio fischeri* and became the first well-described signal and response QS system. Other bacterial systems have since been characterized as QS systems, but they are genetically and mechanistically unrelated to LuxR-LuxI type systems, and seemingly represent examples of convergent evolution. In this short primer, we focus on the canonical LuxR-LuxI type of QS.

**Fig. 1.**
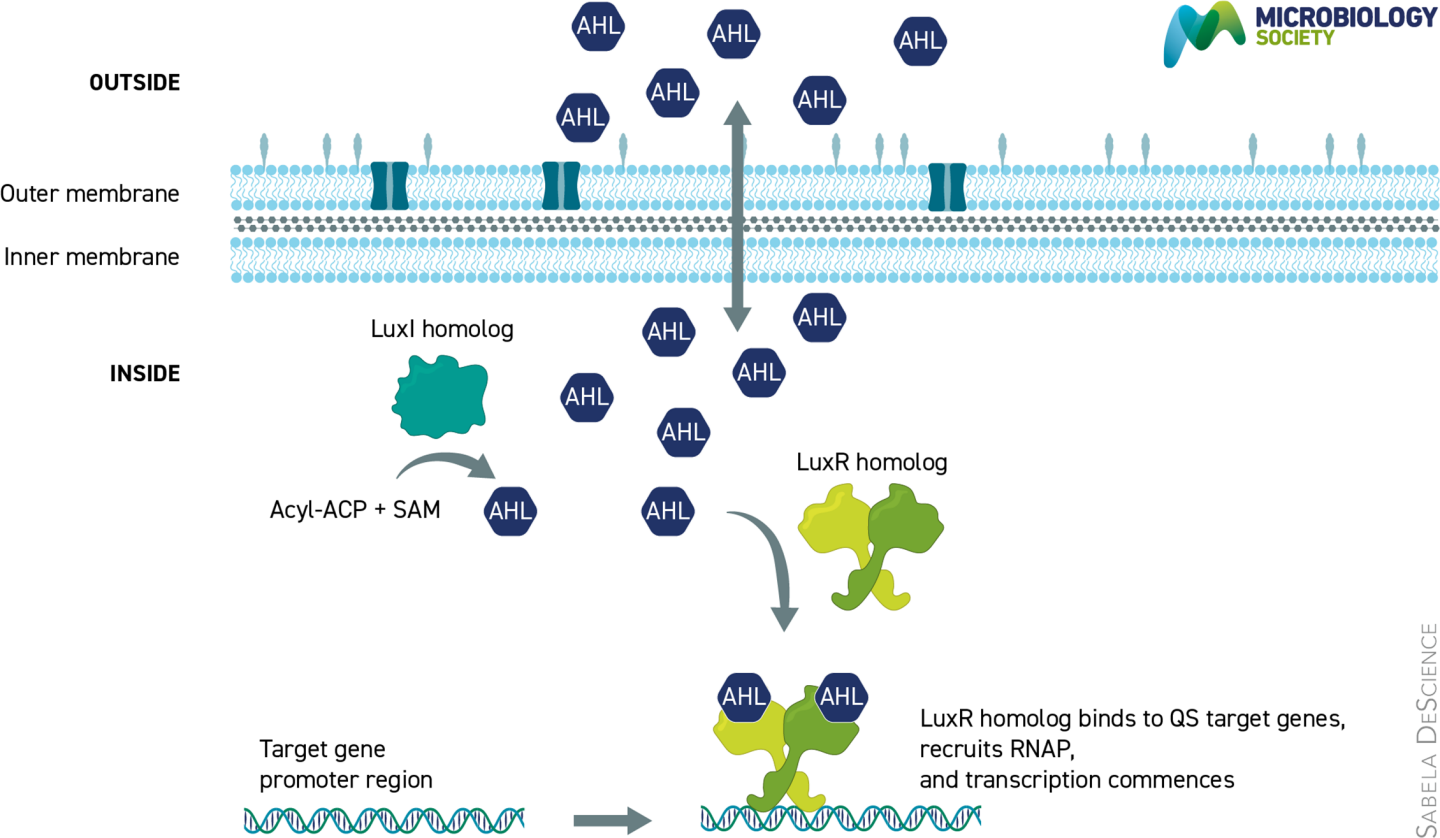
Stylized cartoon of a generic LuxI-LuxR-type QS circuit. A monomeric LuxI homolog catalyses the synthesis of an AHL from an activated organic acid and SAM. The AHL can diffuse in and out of the Gram-negative cell. When the AHL reaches a critical concentration, it can bind to a LuxR homolog and stabilize the active dimeric protein. The AHL-bound LuxR can bind to QS activated promoters and when other conditions are met it recruits RNA polymerase (RNAP) and transcription commences.

In the early 1970s, a phenomenon called autoinduction was shown to control production of bioluminescence by *V. fischeri *. When grown in broth, cells did not produce light until late logarithmic growth phase. This delay in light production during early and mid-logarithmic growth could be shortened by supplementing cultures with a small amount of supernatant fluid from a stationary phase culture, which was hypothesized to contain an autoinducer chemical. About ten years later the autoinducer was identified as *N*-3-oxo-hexanoyl homoserine lactone (3OC6-HSL) and was shown to enter and exit cells by passive diffusion. The *V. fischeri* luminescence genes were cloned and directed *Escherichia coli* to produce light in a cell-density dependent fashion. Two linked genes transcribed independently from each other were responsible for the autoinduction of luminescence; *luxI*, which codes for an enzyme responsible for 3OC6-HSL production, and *luxR*, which codes for a 3OC6-HSL-responsive transcriptional activator of the genes required for light production (Fig. 1).

**Fig. 2.**
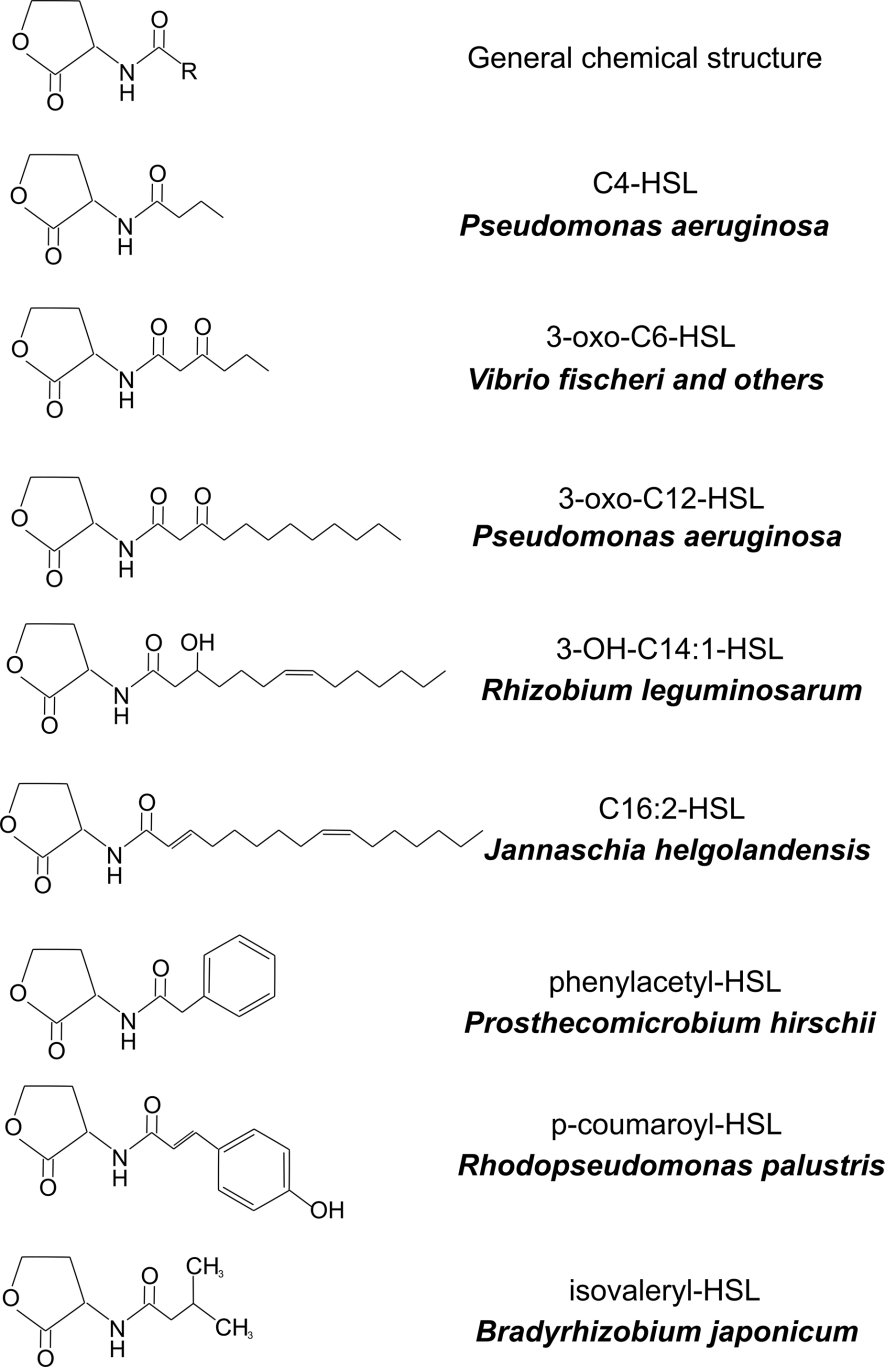
Some examples of AHL quorum sensing signals. The generalized structure of a homoserine lactone with an R group is shown at the top. R is any organic acid. The structures below are compounds produced by the bacteria indicated in the figure.

During this period, we understood how *V. fischeri* controlled light production in a cell density-dependent manner, but it was not until the early 1990s that this type of genetic regulation attracted much attention among microbiologists. In that intervening decade there was a technological leap in DNA sequencing and analysis capabilities. The leap led to the discovery of *luxR* homologs in a number of *Proteobacteria* other than *V. fischeri *. Furthermore, other species of Gram-negative bacteria were shown to produce 3OC6-HSL or acylhomoserine lactones (AHLs) with acyl tails of different structures and lengths (Fig. 2). These findings led to the coining of the term ‘quorum sensing’ and sparked the revolution of the field of bacterial cell-to-cell communication. A quorum was defined as a ‘minimum behavioural unit’ and LuxR-LuxI type systems provide an effective though not unique way for bacteria to take a census of their numbers. The interest was fueled by the fact that LuxR-LuxI-like systems were shown to control virulence in certain human and plant pathogens.

## Mechanistic details of luxi-luxr-dependent qs

There have now been thousands of *luxR-luxI* type genes identified in species of α, β, γ and δ-* Proteobacteria* and in some *Nitrospirota *. Quite often, but not always, cognate *luxR* and *luxI* like genes are adjacent, or at least in close proximity to each other. There are also well documented cases of orphan or solo LuxR homologs, which do not have a cognate LuxI homolog. Some but not all solos can respond to AHLs produced by other bacterial species. The threshold levels of signals for most of the circuits studied are in the range of five to 50 nano-molar, but information is fragmented. Some systems like those of certain members of the genus *Bradyrhizobium* can respond to pico-molar levels, and some other systems require micro-molar levels of signal.

There is extensive diversity among AHL signals, and the majority characterized to date have fatty acyl tails. The chain lengths range from four to 20, and there can be assorted decorations or modification to the fatty acyl tail. Several more recently described AHLs have other types of organic acids as tails, for example aromatic acids or amino acids (Fig. 2). The substrates for these enzymes are in all cases S-adenosyl-methionine (SAM) and an ‘activated’ organic acid. We now understand that there are two subclasses of LuxI-like AHL synthases. One subclass uses fatty acyl-acyl carrier protein (acyl-ACP) substrates from fatty acid biosynthesis as acyl donors, and the other uses acyl-coenzyme A (acyl-CoA) substrates from organic acid degradation pathways or other metabolic pathways with acyl-CoA intermediates. The acyl-CoA subclass has been less-well studied than the acyl-ACP subclass. It is the acyl-CoA enzymes that can produce the more exotic QS signals like those with aromatic acid side chains. Further study of this subclass will certainly lead to the discovery of as yet undescribed AHLs.

Most LuxR-like proteins studied to date are homodimeric transcription activators that can bind to promoter elements of QS-dependent genes when bound to their cognate AHL. A few repressor LuxR-like proteins have also been characterized. These transcription factors bind to specific promoters in the absence of sufficient AHL(s) and block transcription. Sufficient cognate signal therefore derepresses the affected genes. Cellular levels of LuxR homologs are tightly regulated, and in at least one case, artificially high levels of a LuxR-like transcriptional activator results in a ‘short circuit.’

Transcriptomic studies have revealed that some QS systems control expression of hundreds of genes (e.g. *Pseudomonas aeruginosa *) and others control only a handful of mainly linked genes. *V. fischeri* for example, employs LuxR and LuxI to activate the linked six-gene *lux* operon and just a few other *luxI*-linked genes. AHL-dependent QS therefore activates different genes in different bacterial species. It is not uncommon that AHLs activate expression of biosynthetic gene clusters, genes involved in virulence of pathogens like *P. aeruginosa *, and biofilm development. It is important to note that often, but not always, the *luxI* homolog itself is among the genes activated by quorum sensing. This leads to a positive autoregulatory loop that results in a rapid switch from no induction of QS-activated genes to full activation.

## Luxr-luxi-type qs and host interactions

As mentioned above, the first such system to be described was that of *V. fischeri. V. fischeri* QS was immediately considered as a cell-cell communication system, which allowed this species to discriminate between its low population seawater habitat and its high-density existence in the light organs of certain fish and squid species. Light production is an energetically costly process and likely not beneficial in a low-nutrient free-living environment. It is however beneficial and mutualistic in the light organ of a squid. It has since been shown that other host-associated bacteria employ AHL-dependent QS to control expression of virulence genes, and that QS mutants are often impaired in virulence. This is true in human pathogens, for example *P. aeruginosa *, and in plant pathogens, for example *Pantoea carotovora*.

While many studies have demonstrated that QS mutants are often less virulent than their wild-type counterparts in a range of infection models, we still have little understanding of the role of QS in human infection. Such studies are hindered by obvious limitations in human subject research, particularly the inherent difficulties of obtaining and working with human-derived samples. We often assume that QS is important for a number of different human infections such as those found in the cystic fibrosis (CF) lung or in chronic wounds, but this might not always be the case, because QS mutants are often isolated from chronic infection. Indeed, many chronic infections contain heterogenous mixtures of QS positive and QS mutants that have at least partially inactivated QS circuits, and a key challenge in the future is to understand the functional consequences for the bacteria of these mixed populations and the overall impact on the host.

## Sociomicrobiology and quorum sensing

For LuxR-LuxI like systems the QS hypothesis can be stated as follows: ‘Acyl-homoserine lactone QS can serve as a communication system allowing a bacterial species to express genes useful at high population densities only when the cells are at a sufficiently high population density’. This seems evident in the case of *V. fischeri *. The light produced by a few cells is not visible to any known biological detection system, but the light produced by a large group of cells is highly visible. Such behaviours lead us to the idea that QS is a ‘social’ trait, but in principle, QS could be non-social by allowing individual cells to sense changes in their physical environment (this has been termed diffusion sensing). However, it is now well established that in certain environments, QS is social and that, at the population level, QS regulates the production of what we term extracellular public or common goods. These goods (e.g. extracellular proteases) directly benefit the producing cell, but they also provide indirect social benefits to surrounding cells. Because the production of them is costly, these behaviours could potentially be exploited by non-producing ‘cheats’, creating bacterial social dilemmas. AHL-dependent QS has now been shown to be exploitable by cheats both in laboratory cultures and animal models of infection. To maintain QS-dependent cooperation, kin selection has been proposed as an evolutionary strategy, because bacteria use clonal reproduction and the interactions involved in QS are relatively local, meaning that the bacteria involved in QS are likely to be closely related. Other genetic mechanisms employed by bacteria such as the ‘policing’ of cheats has also been demonstrated *in vitro* to maintain AHL-dependent QS.

## Quorum sensing is an integrated component of cellular regulatory networks

We have not yet discussed the fact that many bacteria have multiple *luxR-luxI* type systems. These systems can influence each other, sometimes in a hierarchical manner; when one of the systems turns on another. In some cases, there is crosstalk, when the signal for one QS system can interact with the receptor of another system. In other cases, there is virtually no signal overlap between the systems in a bacterial species. There seems to be a misconception that a sufficient concentration of the cognate AHL for any particular LuxI-LuxR like circuit is a trigger for expression of QS-dependent genes. We have known from the earliest days of research on *V. fischeri* autoinduction that this is not the case, and this has been re-enforced by countless subsequent publications on many different AHL-dependent QS systems. For *V. fischeri,* addition of the cognate 3OC6-HSL signal to the culture broth at the time of inoculation shortens the lag in induction of luminescence but it does not eliminate it. A sufficient concentration if 3OC6-HSL is required but is not sufficient for activation of the luminescence genes. Other conditions must also be met.

## Looking ahead

We can say that there is now a quorum of researchers who work on various AHL quorum sensing systems in many different Gram-negative species. In a relatively short period of about 30 years from the time the term ‘quorum sensing’ was given to LuxR-LuxI like regulatory circuits, the field has blossomed. This is important because there are still many important questions to be answered and there are many research areas that will continue to bear fruit given sufficient focus. We now have a wealth of knowledge to enable development of QS applications. Already there are applications in the realm of wastewater treatment. There has been a focus on using QS circuits in synthetic biology. A key question, which is the focus of considerable activity is whether we can utilize QS as an anti-virulence target of pathogens to treat infections? We know that LuxR-LuxI like regulation can serve as a cell density-dependent gene expression circuit that coordinates cooperative activities in some bacteria in some environments. Can such circuits be adapted to serve other purposes? There has been some work to suggest this possibility. Much work has focused on QS in high cell density populations of a ‘single’ species, for example the light organs of certain marine animals, and *P. aeruginosa* lung infections. There is much to discover about QS in the context of polymicrobial habitats (both environmental and in host association). We also know little about how the diversity of signals evolved. This is an area ripe for future experimental evolution work. Future studies could also focus on QS in microbial communities at the single-cell level, using new transcriptomic techniques, which will allow us to understand more clearly how individual cells signal and respond within a population.

## Further reading

1. Azimi, S., Klementiev, A. D., Whiteley, M. and Diggle, S. P. (2020) Bacterial quorum sensing during infection. *Annual Review of Microbiology*. 74 : 201–219.

2. Bainton, N. J., Bycroft, B. W., Chhabra, S. R., Stead, P., Gledhill, L., Hill, P. J., Rees, C. E., Winson, M. K., Salmond, G. P., Stewart, G. S. and Williams, P. (1992) A general role for the lux autoinducer in bacterial cell signalling: control of antibiotic biosynthesis in Erwinia. *Gene*. 116 : 87–91.

3. Davies, D. G., Parsek, M. R., Pearson, J. P., Iglewski, B. H. and Greenberg, E. P. (1998) The involvement of cell-to-cell signals in the development of a bacterial biofilm. *Science*. 280 : 295–298.

4. Diggle, S. P., Griffin, A. S., Campbell, G. S. and West S. A. (2007) Cooperation and conflict in quorum sensing bacterial populations. *Nature*. 450 : 411–414.

5. Engebrecht, J., Nealson, K. and Silverman, M. (1983) Bacterial bioluminescence: isolation and genetic analysis of functions from *Vibrio fischeri *. *Cell*. 32 : 773–781.

6. Fuqua, W. C., Winans, S. C. and Greenberg, E. P. (1994) Quorum sensing in bacteria: The LuxR-LuxI family of cell density-dependent transcriptional regulators. *Journal of Bacteriology*. 176 : 269–275.

7. Mukherjee, S. and Bassler, B. L. (2019) Bacterial quorum sensing in complex and dynamically changing environments. *Nature Reviews Microbiology*. 17 : 371–382.

8. Redfield, R. J. (2002) Is quorum sensing a side effect of diffusion sensing? *Trends in Microbiology*. 10 : 365–370.

9.Welsh, M. A. and Blackwell, H. E. (2016) Chemical probes of quorum sensing: from compound development to biological discovery. *FEMS Microbiology Reviews*. 40 : 774–794.

10. Whiteley, M., Diggle, S. P. and Greenberg, E. P. (2017) Progress in and promise of bacterial quorum sensing research. *Nature*. 551 : 313–320.

